# Predictors of Coronary Collateral Circulation in Patients with Acute ST-segment Elevation Myocardial Infarction: A Nomogram-based Approach

**DOI:** 10.31083/RCM26477

**Published:** 2025-04-16

**Authors:** Hongxia Shao, Wenling Zhao, Zhao Li, Xingchen Song, Ruifeng Liu

**Affiliations:** ^1^Department of Cardiology, The People’s Hospital of Dangshan County, 235300 Suzhou, Anhui, China; ^2^Department of Cardiology, Beijing Friendship Hospital, Capital Medical University, 100050 Beijing, China

**Keywords:** coronary collateral circulation, acute ST-segment elevation myocardial infarction, predictors, nomogram, Gensini score

## Abstract

**Background:**

Coronary collateral circulation (CCC) is a crucial protective mechanism in acute myocardial infarction. This study aimed to identify early predictors of CCC in patients with acute ST-segment elevation myocardial infarction (STEMI) and develop a nomogram for predicting its presence.

**Methods:**

We conducted a retrospective study of STEMI patients admitted to the Beijing Friendship Hospital from January 2015 to December 2023. Patients with CCC, as confirmed by coronary angiography, were matched 1:3 with those without CCC based on the date of admission. We compared baseline characteristics, laboratory parameters, coronary features, and in-hospital outcomes between the two groups. Variable selection was performed using least absolute shrinkage and selection operator (LASSO) regression analysis, followed by univariable and multivariable logistic regression analyses to identify independent predictors of CCC. A nomogram was constructed based on significant predictors and was validated through receiver operating characteristic (ROC) curve analysis, calibration curves, and decision curve analysis.

**Results:**

A total of 668 patients with STEMI were included in the study (501 without CCC and 167 with CCC). Patients with CCC had a higher prevalence of right coronary artery (RCA) closure and multi-vessel disease, as well as elevated inflammatory markers and altered coagulation parameters. Multivariable logistic regression analysis identified a history of coronary heart disease (CHD), osmolality, levels of fibrinogen, and left anterior descending (LAD) artery closure, left circumflex (LCX) artery closure, and RCA closures, as well as the Gensini score, were independent predictors of CCC. The nomogram incorporating these predictors demonstrated good discrimination and calibration, indicating an accurate prediction of the presence of CCC.

**Conclusions:**

History of CHD, osmolality, levels of fibrinogen, LAD, LCX, and RCA closures, as well as the Gensini score, are independent predictors of CCC in patients with STEMI. The developed nomogram offers a clinically useful tool for identifying patients likely to have CCC, potentially aiding in personalized treatment strategies.

## 1. Introduction

Coronary heart disease (CHD) remains a leading cause of morbidity and mortality worldwide [1]. Among its various manifestations, acute ST-segment elevation myocardial infarction (STEMI) stands out as particularly severe, causing significant myocardial damage and impaired cardiac function [2]. The development of a well-functioning coronary collateral circulation (CCC) has emerged as a crucial protective mechanism against myocardial ischemia in CHD patients [3, 4]. CCC consists of a network of small arterial connections that can form between different coronary artery territories, offering an alternative blood supply to the myocardium distal to an occluded or severely stenosed coronary artery [5, 6]. A well-developed CCC has been associated with smaller infarct sizes, improved left ventricular function, lower mortality rates, and a reduced incidence of malignant arrhythmias in patients with STEMI [7, 8]. However, the development of CCC varies widely among individuals, and the factors influencing its formation remain poorly understood.

Previous studies have highlighted several clinical, angiographic, and genetic factors that may influence the development of CCC, including age, diabetes, hyperlipidemia, and specific genetic polymorphisms [9, 10]. However, those studies often lacked specificity in their predictors, frequently focusing on isolated factors without considering the complex interplay between multiple clinical variables. For instance, some studies primarily emphasized genetic polymorphisms or single clinical factors such as age or diabetes, without integrating these with angiographic or laboratory data, which are crucial for a holistic understanding of CCC development [11, 12]. Consequently, there is a need for predictive models that incorporate a broader range of clinical data and are validated in varied populations to enhance their utility in clinical practice. This study aims to investigate the clinical, angiographic, and laboratory parameters associated with the presence of CCC in patients with acute STEMI. Additionally, it seeks to develop a predictive nomogram to identify patients at high risk for poor CCC development. By elucidating the determinants of CCC formation, this study may contribute to improved risk stratification and personalized treatment strategies, ultimately improving CCC and improving clinical outcomes in patients with STEMI.

## 2. Materials and Methods

### 2.1 Study Design and Patient Population

**Fig. 1.**
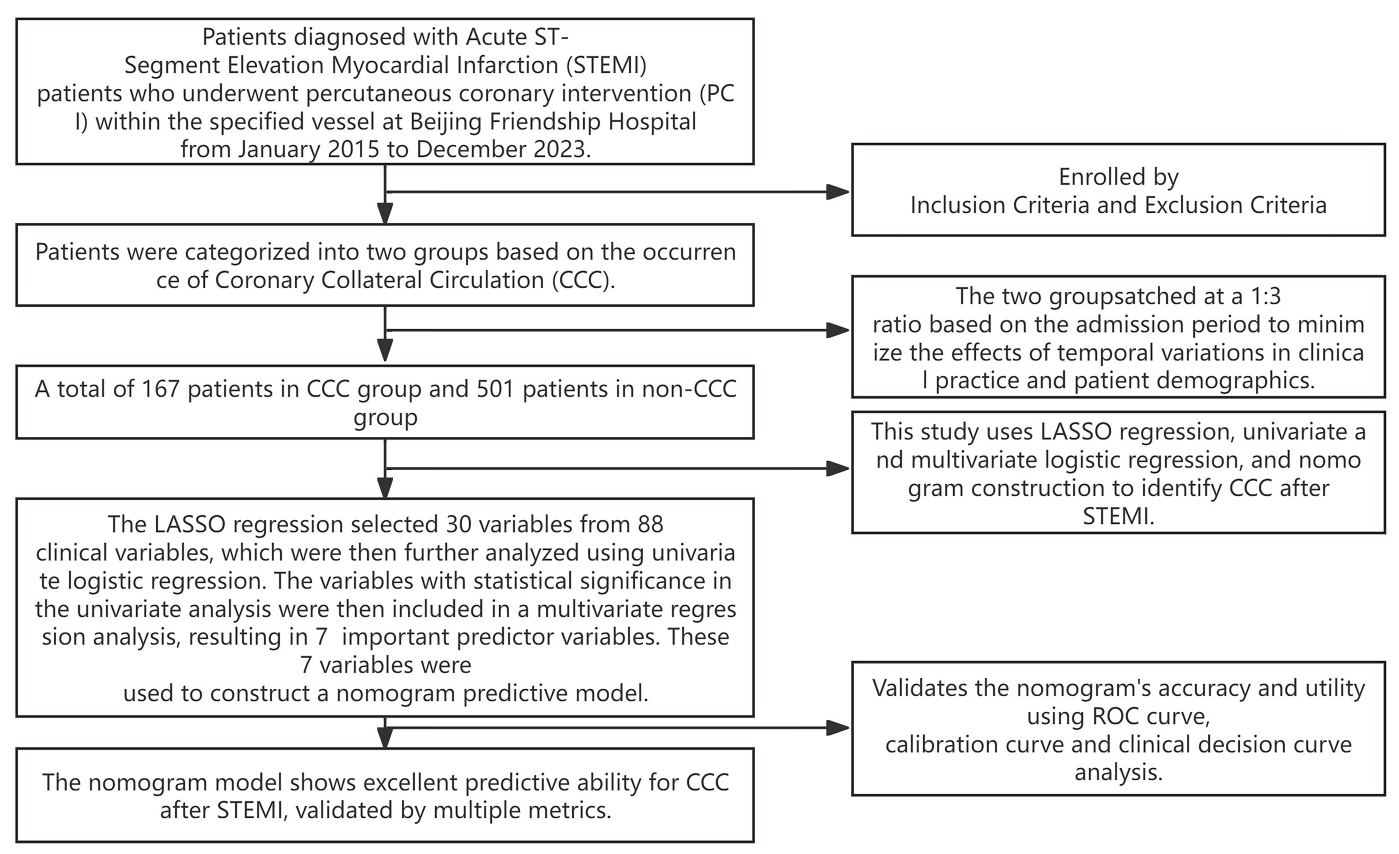
**Flowchart of this retrospective case-control study**. CCC, coronary collateral circulation; LASSO, least absolute shrinkage and selection operator; ROC, receiver operating characteristic.

This single-center, retrospective observational study was conducted at the Beijing Friendship Hospital from January 2015 to December 2023. We included patients diagnosed with STEMI who underwent primary percutaneous coronary intervention (PCI). Patients were categorized into two groups based on the presence or absence of CCC observed during the index PCI procedure. The CCC group comprised patients with angiographically visible CCC (Rentrop grade ≥1), while the non-CCC group included patients without angiographic evidence of CCC (Rentrop grade 0). A 1:3 matched control group was created, matching patients in the non-CCC group to those in the CCC group by the time of admission time. The study protocol was showed in Fig. 1 and it was approved by the Institutional Review Board of the Beijing Friendship Hospital (Approval No. 2018-P2-030-01), these patients were informed during their hospitalization that their medical data might be used for medical research, and their informed consent was obtained.

### 2.2 Inclusion Criteria

The inclusion criteria were as follows: (1) patients aged ≥18 years; (2) patients diagnosed with acute STEMI, diagnosed following guidelines set by the Chinese Society of Cardiology; (3) patients eligible for PCI, having no contraindications, and who underwent either primary PCI or percutaneous transluminal coronary angioplasty (PTCA) within 12 hours after STEMI occurred; (4) patients whose complete angiographic data, including Rentrop collateral grade, were available; and (5) patients whose complete clinical and laboratory data, encompassing cardiovascular risk factors, medical history, symptoms, and biochemical markers, were available.

### 2.3 Exclusion Criteria

The exclusion criteria were as follows: patients with (1) a history of myocardial infarction or previous revascularization with pre-existing collaterals; (2) severe mechanical complications, acute left heart failure, sudden cardiac death, or cardiogenic shock, to avoid potential difficulties for the assessment of CCC; (3) severe valvular or congenital heart diseases, or other structural heart diseases which may affect normal cardiovascular function; (4) malignancy, advanced renal disease, severe infection, severe liver injury, or other severe comorbidities (5) incomplete coronary angiography or clinical data, and (6) patients who did not provide informed consent.

### 2.4 Data Collection

Baseline demographic, clinical, laboratory, and angiographic data were collected from the medical records of the patients. This data included age, gender, cardiovascular risk factors (such as hypertension, diabetes, dyslipidemia, and smoking status), history of prior myocardial infarction, culprit vessel and Rentrop collateral grade. Laboratory parameters, including complete blood count, lipid profile, and cardiac biomarkers, were also recorded.

### 2.5 Assessment of CCC

CCC was assessed by two experienced interventional cardiologists who were blinded to the clinical data of the patients. The degree of CCC was graded using the Rentrop classification system, which ranges from “zero” (no visible collaterals) to “three” (complete filling of the epicardial vessel distal to the occlusion) [6].

### 2.6 Statistical Analysis

Continuous variables were presented as either mean ± standard deviation, or median (interquartile range), depending on their distribution. Categorical variables were presented as frequencies and percentages. Differences between the CCC and non-CCC groups were analyzed using the student’s *t*-test, Mann-Whitney U test, or chi-square test, as appropriate.

We performed least absolute shrinkage and selection operator (LASSO) regression analysis on the collected variables to identify the most relevant predictors of CCC. Variables selected by LASSO regression analysis were then subjected to univariable and multivariable logistic regression analyses to determine the independent predictors of CCC. Based on the results of the multivariable analysis, a nomogram was constructed to visually predict the probability of CCC.

The performance of the nomogram was evaluated using receiver operating characteristic (ROC) curve analysis, calibration plots, and decision curve analysis (DCA). The area under the ROC curve (AUC) was calculated to assess the discriminative ability of the nomogram. Calibration plots assessed the agreement between predicted probabilities and observed outcomes, while DCA quantified the net benefits of the nomogram at various threshold probabilities to determine its clinical usefulness.

All statistical analyses were conducted using R software (version 4.4.0, The R Foundation for Statistical Computing, Vienna, Austria). A two-sided *p*-value of <0.05 was considered statistically significant.

## 3. Results

### 3.1 Primary Baseline Characters for Enrolled Patients 

Table 1 shows the baseline characteristics of the 668 enrolled patients, categorized into two groups: 501 patients in the non-CCC group and 167 in the CCC group. The table highlights several key findings between the non-CCC and CCC groups. Notably, the CCC group has a significantly higher percentage of patients with a history of CHD (26.95% vs. 13.97%) and higher fibrinogen levels (median 3.00 g/L vs. 2.84 g/L), with *p*-values of <0.000 and 0.002, respectively. Additionally, the CCC group has a slightly higher average body mass index (BMI) (25.86 kg/m^2^ vs. 25.13 kg/m^2^) and lower osmolality (median 287.50 mOsm/kg vs. 289.30 mOsm/kg), with *p*-values of 0.021 for both. These findings suggest that CHD history, fibrinogen levels, BMI, and osmolality may be associated with the development of CCC. For additional relevant patient information, please refer to the **Supplementary Materials**.

**Table 1.** **Primary baseline characters for enrolled subjects**.

Characteristic	non-CCC group, n = 501	CCC group, n = 167	Z/χ^2^/t	*p*-value
Age (years)	61.00 (54.00, 71.00)	61.00 (54.00, 73.00)	–0.246	0.805
Gender (male, n, %)	383 (76.45%)	134 (80.24%)	1.030	0.310
CHD history (n, %)	70 (13.97%)	45 (26.95%)	14.793	<0.001
Diabetes (n, %)	123 (24.55%)	33 (19.76%)	1.606	0.205
Hypertension (n, %)	268 (53.49%)	102 (61.08%)	2.916	0.088
Smoking (n, %)	268 (53.49%)	84 (50.30%)	0.512	0.474
Alcohol consumption (n, %)	201 (40.12%)	63 (37.72%)	0.031	0.583
BMI (kg/m^2^)	25.13 ± 3.57	25.86 ± 3.45	–2.315	0.021
ALT (U/L)	80.40 (30.00, 178.00)	89.00 (37.00, 174.00)	–0.209	0.834
Creatinine (µmol/L)	82.00 (73.10, 90.30)	84.20 (75.00, 93.90)	–1.629	0.073
Blood urea nitrogen (mmol/L)	5.23 (4.09, 6.53)	5.46 (4.45, 6.78)	–1.911	0.056
Total cholesterol (mmol/L)	4.50 (3.85, 5.12)	4.45 (3.84, 5.21)	–0.431	0.666
Triglycerides (mmol/L)	1.41 (1.02, 1.89)	1.39 (1.07, 1.94)	–0.426	0.670
High density lipoprotein cholesterol (mmol/L)	1.05 (0.91, 1.20)	1.04 (0.89, 1.19)	–0.756	0.450
Low density lipoprotein cholesterol (mmol/L)	2.64 (2.19, 3.08)	2.64 (2.14, 3.08)	–0.299	0.765
Fibrinogen (mg/dL)	2.84 (2.29, 3.27)	3.00 (2.38, 3.71)	–3.073	0.002
Beta blockers on admission (n, %)	27 (5.39%)	16 (9.58%)	3.654	0.056
CCB on admission (n, %)	136 (27.15%)	48 (28.74%)	0.160	0.689
Osmolality (mOsm/kg)	289.30 (283.10, 294.20)	287.50 (280.20, 292.50)	–2.314	0.021
Lactic acid (mmol/L)	2.13 (1.82, 2.49)	2.13 (1.77, 2.50)	–0.367	0.713
Antiplatelet drugs before admission (n, %)	72 (14.37%)	24 (14.37%)	0.000	1.000
RAAS inhibitor (n, %)	65 (12.97%)	22 (13.17%)	0.004	0.947
Statins (n, %)	50 (9.98%)	14 (8.38%)	0.369	0.544
Diuretics on admission (n, %)	6 (1.20%)	4 (2.40%)	0.541	0.462

Abbreviations: CHD, coronary heart disease; BMI, body mass Index; ALT, alanine aminotransferase; CCB, calcium channel blocker; RAAS inhibitor, renin-angiotensin-aldosterone system inhibitor.

### 3.2 Coronary Characteristics and In-hospital Prognosis

Table 2 showed, in the CCC group, a substantial proportion of patients had good collateral blood flow (78.44%), while none in the non-CCC group did. The CCC group had higher rates of left anterior descending (LAD), left circumflex (LCX), and right coronary artery (RCA) closures, with *p*-values of 0.009, <0.001, and <0.001, respectively. The Gensini score, which reflects the severity of coronary artery disease, was significantly higher in the CCC group (median 116.00) compared to the non-CCC group (median 83.00), with a *p*-value < 0.001. Additionally, admission and peak N-terminal pro B-type natriuretic peptide (NT-proBNP) levels, indicating more severe heart stress, with *p*-values of 0.008 and 0.022, respectively. The use of intra-aortic balloon pump (IABP) was significantly more frequent in the CCC group (6.59% vs. 1.80%), and their hospital stay was longer (median 9 days vs. 8 days), both with *p*-values of 0.004 and <0.001, respectively. There were no significant differences in major adverse cardiac events (MACE), cardiogenic death, recurrent myocardial infarction, cerebral infarction, or cerebral hemorrhage between the groups. For further patient details, please refer to the **Supplementary Materials**.

**Table 2.** **Coronary characteristics and in-hospital prognosis**.

Characteristic		non-CCC group, n = 501	CCC group, n = 167	Z/χ^2^	*p*-value
CCC blood flow					
	No (n%)	501 (0.00)	0.00 (0.00)	668.00	<0.001
	Bad (n%)	0.00 (0.00)	36 (21.56%)		
	Good (n%)	0.00 (0.00)	131 (78.44%)		
LM closure (n, %)		4 (0.80%)	0 (0.00%)	0.335	0.563
LAD closure (n, %)		131 (26.15%)	62 (37.13%)	7.347	0.007
LCX closure (n, %)		46 (9.18%)	39 (23.35%)	22.651	<0.001
RCA closure (n, %)		85 (16.97%)	87 (52.10%)	80.848	<0.001
Gensini socre		83.00 (62.00, 110.50)	116.00 (86.75, 144.00)	–8.643	<0.001
IABP (n, %)		9 (1.80%)	11 (6.59%)	9.896	0.002
stent (n, %)		478 (95.41%)	161 (96.41%)	0.300	0.584
Thrombus aspiration (n, %)		23 (4.59%)	5 (2.99%)	0.795	0.373
Admissio NT-proBNP (pg/mL)		534.00 (131.00, 1930.19)	1032.00 (183.00, 2216.50)	–2.635	0.008
Peak NT-proBNP (pg/mL)		1656.00 (666.00, 3804.00)	2202.00 (1080.50, 4286.50)	–2.282	0.022
CKMB peak (ng/mL)		114.75 (28.90, 201.00)	114.75 (28.55, 242.00)	–0.173	0.863
MYO peak (ng/mL)		150.00 (50.30, 285.00)	171.00 (48.40, 299.00)	–0.498	0.619
TnI peak (ng/mL)		12.50 (3.46, 25.00)	12.64 (4.18, 27.35)	–0.412	0.680
Killip ≥II grade (n, %)		395 (78.84%)	128 (76.65%)	0.355	0.551
Length of hospital stay (Days)		8.00 (6.00, 10.00)	9.00 (7.00, 11.00)	–3.655	0.000
MACE (n, %)		14 (2.79%)	2 (1.20%)	0.768	0.381
Cardiogenic death (n, %)		6 (1.21%)	2 (1.20%)	0.000	1.000
Recurrent myocardial infarction (n, %)		2 (0.40%)	1 (0.60%)	-	1.000
Cerebral infarction (n, %)		3 (5.99%)	1 (0.60%)	0.000	1.000
Cerebral hemorrhage (n, %)		2 (0.40%)	1 (0.60%)	-	1.000

Abbreviations: LM, left main; LAD, left anterior descending; LCX, left circumflex; RCA, right coronary artery; IABP, intra-aortic balloon pump; NT-proBNP, N-terminal pro B-type natriuretic peptide; CKMB, creatine kinase-MB; MYO, myoglobin; TnI, troponin I; MACE, major adverse cardiac events.

### 3.3 LASSO Regression Analysis for Identifying Key Predictors

**Fig. 2.**
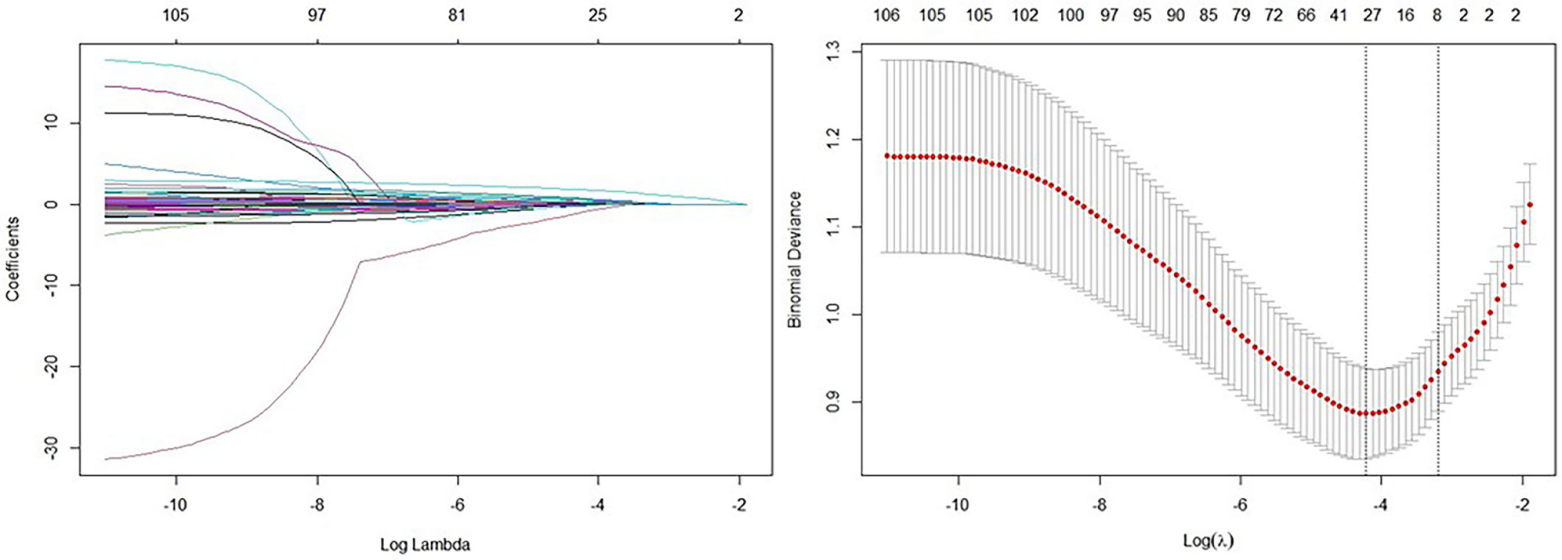
**LASSO regression analysis selecting related item for CCC**. Note: (1) Left: Coefficient profile plot. The coefficient profile plot illustrates the change in the magnitude of each coefficient as the penalty parameter (lambda) increases. As lambda increases, more coefficients are reduced towards zero in a simpler model with fewer predictors. (2) Right: Cross-validation plot from LASSO regression analysis. The cross-validation plot shows the performance of the model, such as mean squared error, for various lambda values. This plot is used to identify the optimal lambda value that minimizes out-of-sample prediction error, achieving a balance between model complexity and predictive accuracy. (3) Using an optimal lambda_min value of 0.01472, 30 variables were selected as significant predictors from 88 items. The selected variables and their coefficients are as follows: age (–9.0062 × 10^-03^), history of CHD (5.4443 × 10^-01^), prior myocardial infarction (3.8011 × 10^-01^), diabetes (–1.1056 × 10^-01^), duration of diabetes (3.4746 × 10^-03^), beta-blockers use on admission (3.3271 × 10^-01^), family history of early-onset CHD (–2.5897 × 10^-02^), family history of ischemic stroke (–2.3142 × 10^-01^), family history of hemorrhagic stroke (1.7612 × 10^-01^), NT-proBNP at admission (2.8717 × 10^-06^), red blood cells (7.3242 × 10^-02^), mean corpuscular hemoglobin (–2.5730 × 10^-02^), ALT (–2.0129 × 10^-04^), globulin (6.3066 × 10^-03^), lactic acid (–1.0391 × 10^-01^), low-density lipoprotein cholesterol (1.0075 × 10^-02^), high-sensitivity C-reactive protein (1.1336 × 10^-03^), potassium (7.1077 × 10^-02^), chloride (–6.0830 × 10^-02^), carbon dioxide (1.0784 × 10^-02^), osmolality (–5.1357 × 10^-03^), prothrombin time (–1.1948 × 10^-02^), levels of fibrinogen (1.0555 × 10^-01^), thyroxine (1.0420 × 10^-02^), LM closure (–9.7422 × 10^-01^), LAD closure (6.5542 × 10^-01^), LCX closure (7.4271 × 10^-01^), RCA closure (1.7897 × 10^+00^), RCA lesion (2.6392 × 10^-01^), and Gensini score (–1.19458 × 10^-02^). These variables were identified as potential predictors of CCC and were further analyzed to assess their relevance in predicting CCC.

LASSO regression analysis (Fig. 2) was used to identify potential predictors of CCC. Using an optimal lambda value, 30 significant predictors were selected from a total of 88 items (As showed in Tables 1,2, and the **Supplementary Materials**). The selected variables included: age, history of CHD, prior myocardial infarction, diabetes, duration of diabetes, use of beta-blockers on admission, family history of early-onset CHD, family history of ischemic stroke, family history of hemorrhagic stroke, NT-proBNP at admission, red blood cells, mean corpuscular hemoglobin, alanine aminotransferase (ALT), globulin, lactic acid, low-density lipoprotein cholesterol, high-sensitivity C-reactive protein, potassium, chloride, carbon dioxide, osmolality, prothrombin time, levels of fibrinogen, thyroxine, left main closure, LAD closure, LCX closure, RCA closure, RCA lesion, and Gensini score. These variables were identified as potential predictors of CCC and were further analyzed to evaluate their relevance in predicting CCC development.

### 3.4 Logistic Regression for Predictors of CCC

Table 3 summarizes the results of both univariable and multivariable logistic regression analyses used to identify predictors of CCC. In the multivariable model, several factors were independently associated with CCC: a history of CHD (OR = 2.129, 95% CI: 1.262–3.590, *p* = 0.005), higher fibrinogen levels (OR = 1.375, 95% CI: 1.119–1.689, *p* = 0.002) and Gensini scores (OR = 1.012, 95% CI: 1.005–1.018, *p* = 0.001), lower osmolality (OR = 0.970, 95% CI: 0.947–0.993, *p* = 0.011), LAD closure (OR = 3.368, 95% CI: 1.889–6.003, *p* < 0.001), LCX closure (OR = 3.434, 95% CI: 1.746–6.752, *p* < 0.001), and RCA closure (OR = 11.156, 95% CI: 6.488–19.182, *p* < 0.001).

**Table 3.** **Logistic regression for predicters of coronary collateral circulation**.

Variables	Crude odds ratio		Adjusted odds ratio	
	OR and 95% CI	*p*-value	OR and 95% CI	*p*-value
Age (years)	1.000 (0.986–1.014)	0.980		
CHD history (n, %)	2.271 (1.485–3.474)	<0.001	2.129 (1.262–3.590)	0.005
Old myocardial infarction (n, %)	2.735 (1.274–5.875)	0.010		
Diabetes (n, %)	0.757 (0.492–1.166)	0.206		
Years of diabetes (years)	1.016 (0.961–1.075)	0.579		
Beta blockers on admission (n, %)	1.860 (0.976–3.545)	0.059		
Family history of early onset CHD (n, %)	0.270 (0.030–2.090)	0.209		
Family history of ischemic stroke (n, %)	0.441 (0.183–1.062)	0.068		
Family history of hemorrhagic stroke (n, %)	1.732 (0.898–3.342)	0.101		
Admissio NT-proBNP (pg/mL)	1.000 (1.000–1.000)	1.000		
Red blood cells (10^12^/L)	1.216 (0.891–1.658)	0.218		
Mean orpuscular hemoglobin (pg)	0.920 (0.842–1.007)	0.069		
ALT (U/L)	1.000 (0.999–1.001)	0.805		
Globulin (g/dL)	1.053 (1.008–1.101)	0.021		
Lactic acid (mmol/L)	0.941 (0.716–1.239)	0.667		
Low density lipoprotein cholesterol (mmol/L)	1.160 (0.920–1.450)	0.211		
High sensitivity C-reactive protein (mmol/L)	1.030 (1.010–1.040)	0.001		
Potassium (mmol/L)	1.397 (0.866–2.254)	0.170		
Chloride (mmol/L)	0.928 (0.887–0.971)	0.001		
Carbon dioxide (mmol/L)	1.002 (0.933–1.075)	0.965		
Osmolality (mOsm/kg)	0.996 (0.976–1.016)	0.707	0.970 (0.947–0.993)	0.011
Prothrombin time activity (%)	0.982 (0.970–0.994)	0.004		
Fibrinogen (g/L)	1.345 (1.129–1.603)	0.001	1.375 (1.119–1.689)	0.002
Thyroxine (g/L)	1.014 (1.005–1.023)	0.002		
LM closure (n, %)	0.001 (0.000–Inf)	0.976		
LAD closure (n, %)	1.668 (1.150–2.419)	0.007	3.368 (1.889–6.003)	<0.001
LCX closure (n, %)	3.014 (1.885–4.820)	<0.001	3.434 (1.746–6.752)	<0.001
RCA closure (n, %)	5.322 (3.629–7.805)	<0.001	11.156 (6.488–19.182)	<0.001
RCA lesion (n, %)	4.072 (2.441–6.795)	<0.001		
Gensini score	1.021 (1.016–1.026)	<0.001	1.012 (1.005–1.018)	0.001

OR, odds ratio.

### 3.5 Nomogram for Predicting CCC in Patients with STEMI

**Fig. 3.**
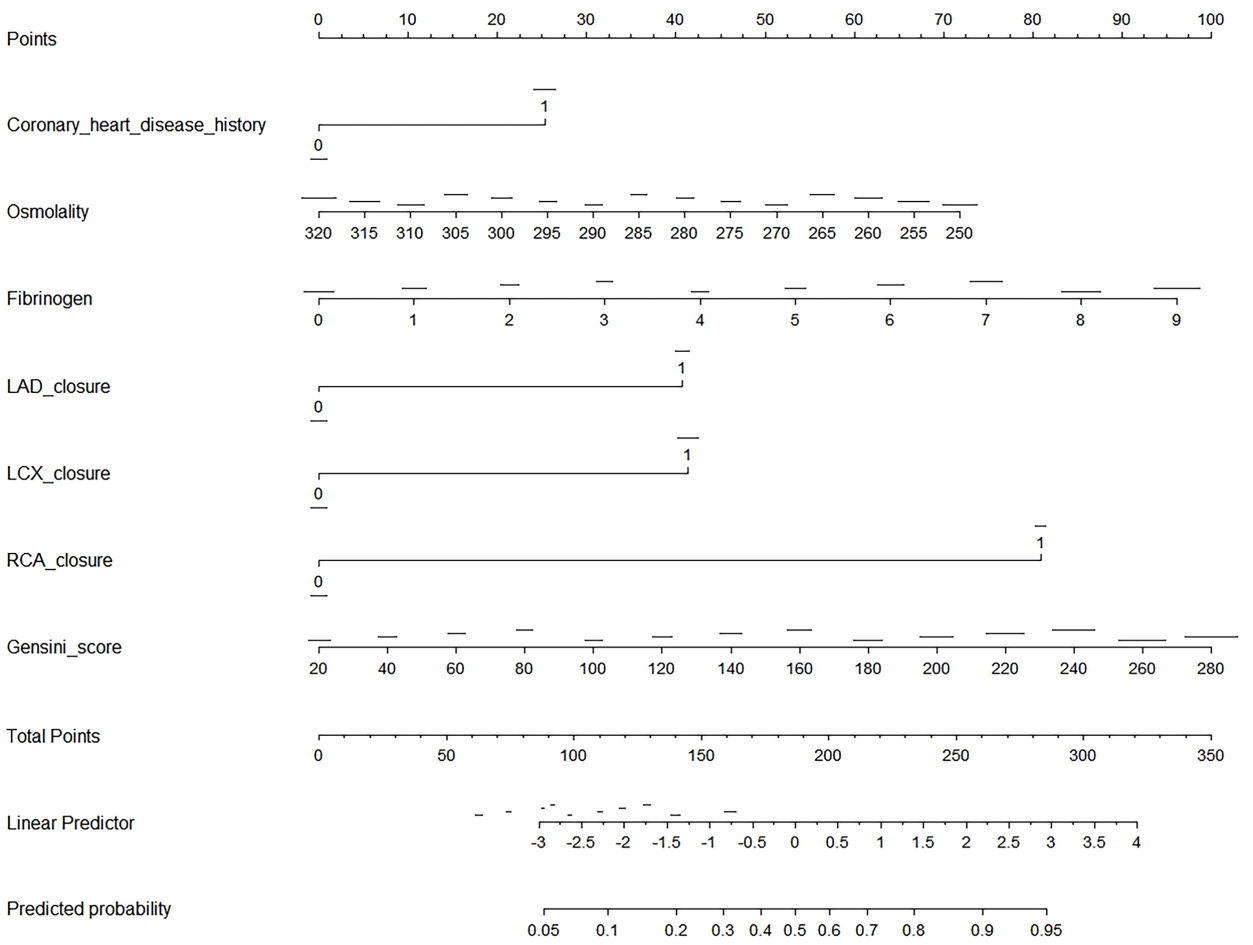
**Nomogram for predicting CCC in patients with STEMI**. This image is a nomogram designed to predict the probability of an outcome based on several clinical variables. To use the nomogram, first identify the variables, which include coronary heart disease history (binary: 0 or 1), osmolality (ranging from 250 to 320), fibrinogen (0 to 9), LAD closure (binary: 0 or 1), LCX closure (binary: 0 or 1), RCA closure (binary: 0 or 1), and Gensini score (ranging from 20 to 280). For each variable, locate its value on the corresponding scale and draw a vertical line up to the “Points” scale at the top, recording the number of points for each variable. Next, sum the points from all variables to get a total score, which can range from 0 to 350. Use the “Total Points” scale to find the corresponding value on the “Linear Predictor” scale, which ranges from –3 to 4. Finally, convert the linear predictor value to a predicted probability using the “Predicted Probability” scale, which ranges from 0.05 to 0.95. This nomogram provides a visual and quantitative method to assess the likelihood of an outcome by integrating multiple clinical factors into a single predictive model.

Fig. 3 presents a nomogram, a graphical statistical tool designed to estimate the probability of CCC in patients with STEMI. This nomogram incorporates several variables, including history of CHD, osmolality, levels of fibrinogen, and LAD, LCX, and RCA closures, as well as the Gensini score. Each variable is assigned a specific point value based on its measurement, which is then totaled to generate an overall score. This total score is used to determine the predicted probability of CCC, as indicated on the nomogram’s linear predictor scale at the bottom.

### 3.6 ROC Curve Analysis for Nomogram Validation

**Fig. 4.**
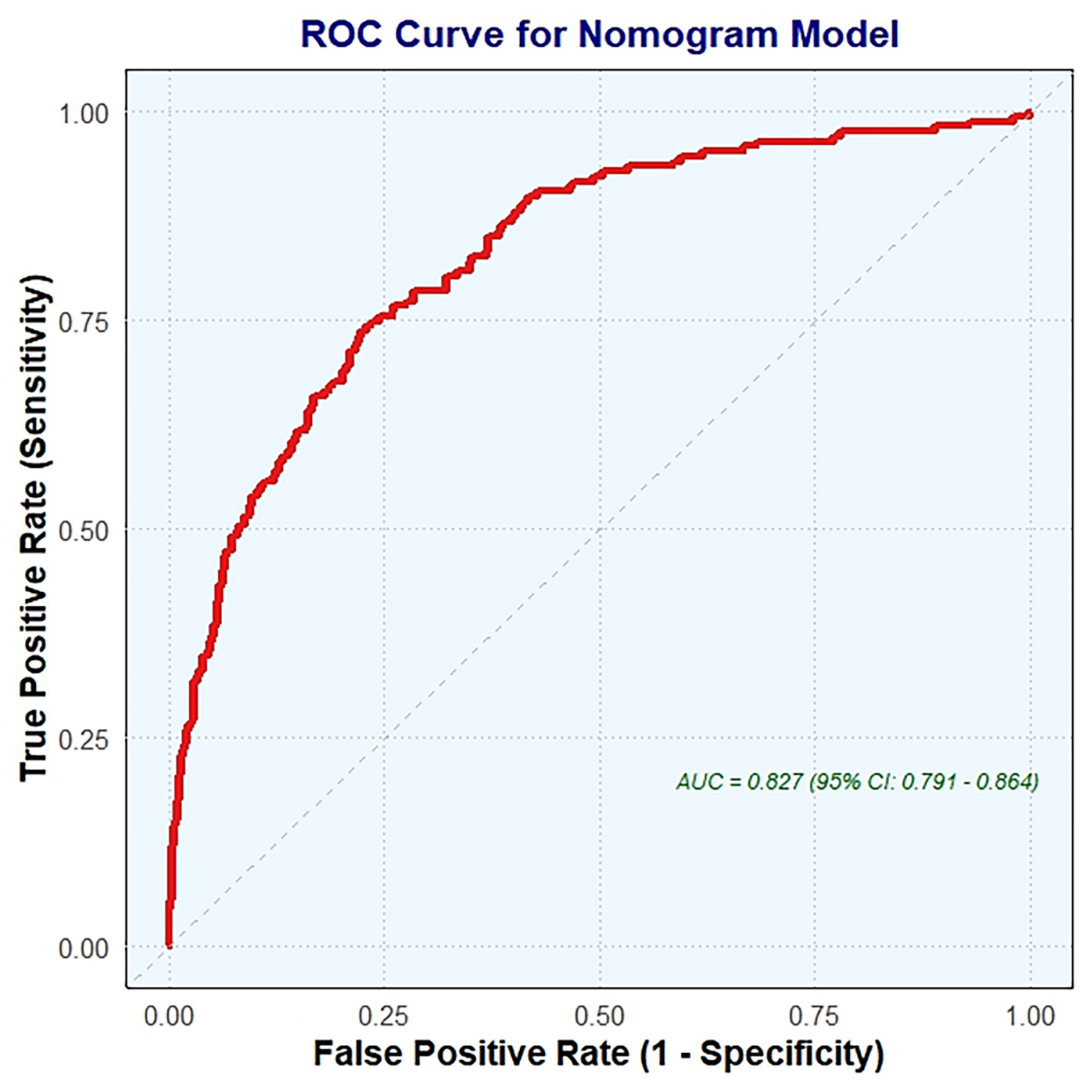
**ROC curve analysis for nomogram validation**. ROC, receiver operating characteristic; AUC, the area under the ROC curve.

The nomogram (Fig. 4) was validated using ROC curve analysis. The optimal cutoff value for the nomogram was identified as 153.05, which resulted in a sensitivity of 0.749 and a specificity of 0.236. This indicates that the nomogram correctly identified 74.9% of patients with CCC but only 23.6% of patients without CCC. The positive predictive value (PPV) was 0.514, indicating that 51.4% of patients scoring above the cutoff actually had CCC. Conversely, the negative predictive value (NPV) was 0.901, showing that 90.1% of patients scoring below the cutoff did not have CCC. The Youden index, which measures the ability of a nomogram to discriminate between patients with and without CCC, was –0.016. The AUC was 0.827 (95% CI, 0.791–0.864), indicating good overall predictive accuracy of the nomogram.

### 3.7 Calibration Curve Analysis for Nomogram Validation

**Fig. 5.**
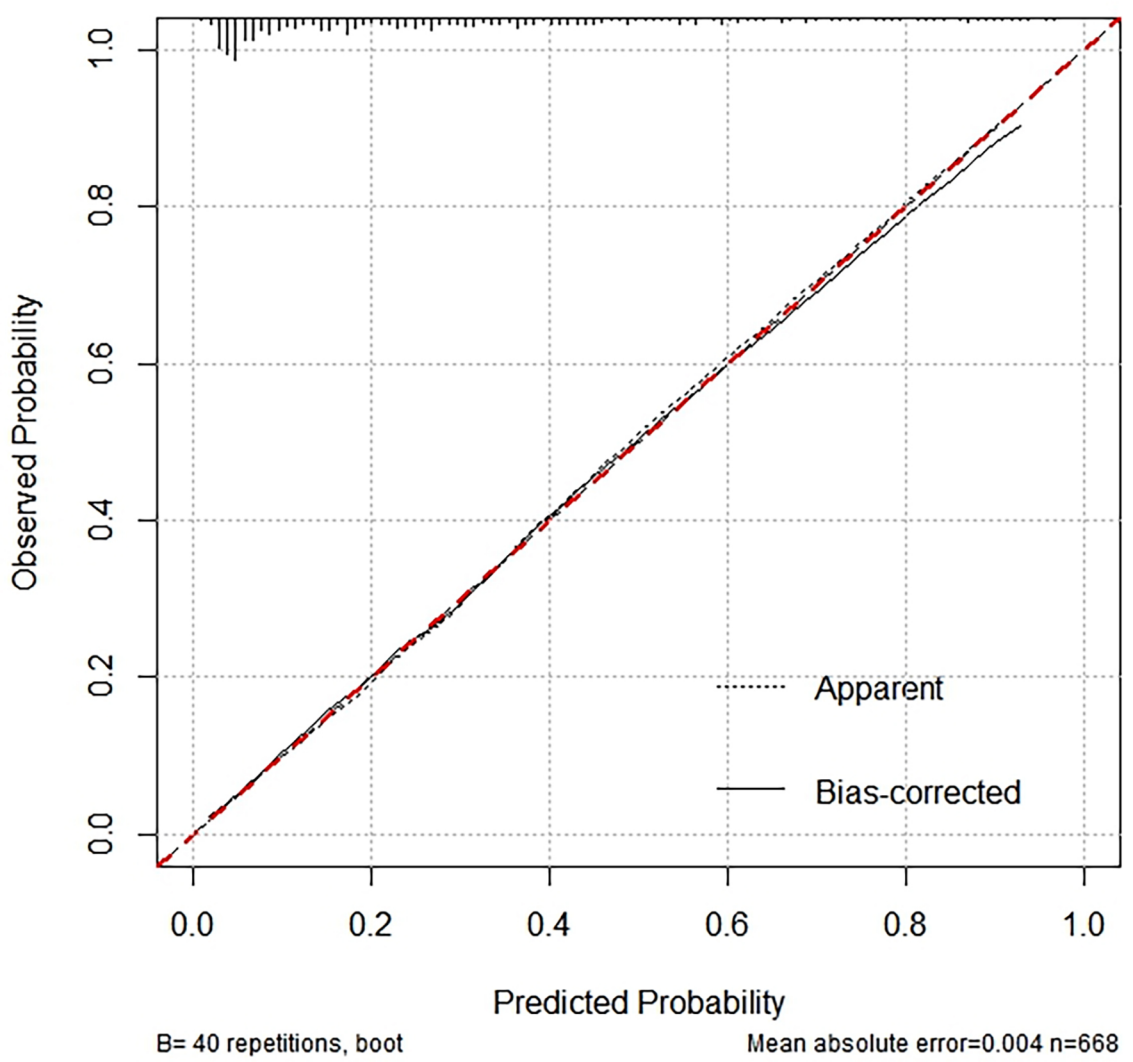
**Calibration curve analysis for nomogram validation**.

The performance of the nomogram was evaluated using a calibration curve (Fig. 5), which compares the predicted probabilities from the nomogram with the actual observed frequencies. The test produced a Hosmer-Lemeshow statistic of 0.050 with 10 degrees of freedom, resulting in a *p*-value of 1.000. It indicates no statistically significant difference between the predicted probabilities and the observed frequencies. Consequently, the calibration curve demonstrates excellent agreement, suggesting that the nomogram is well-calibrated and provides accurate predictions of the presence of CCC across various risk levels.

### 3.8 DCA for Nomogram Validation

**Fig. 6.**
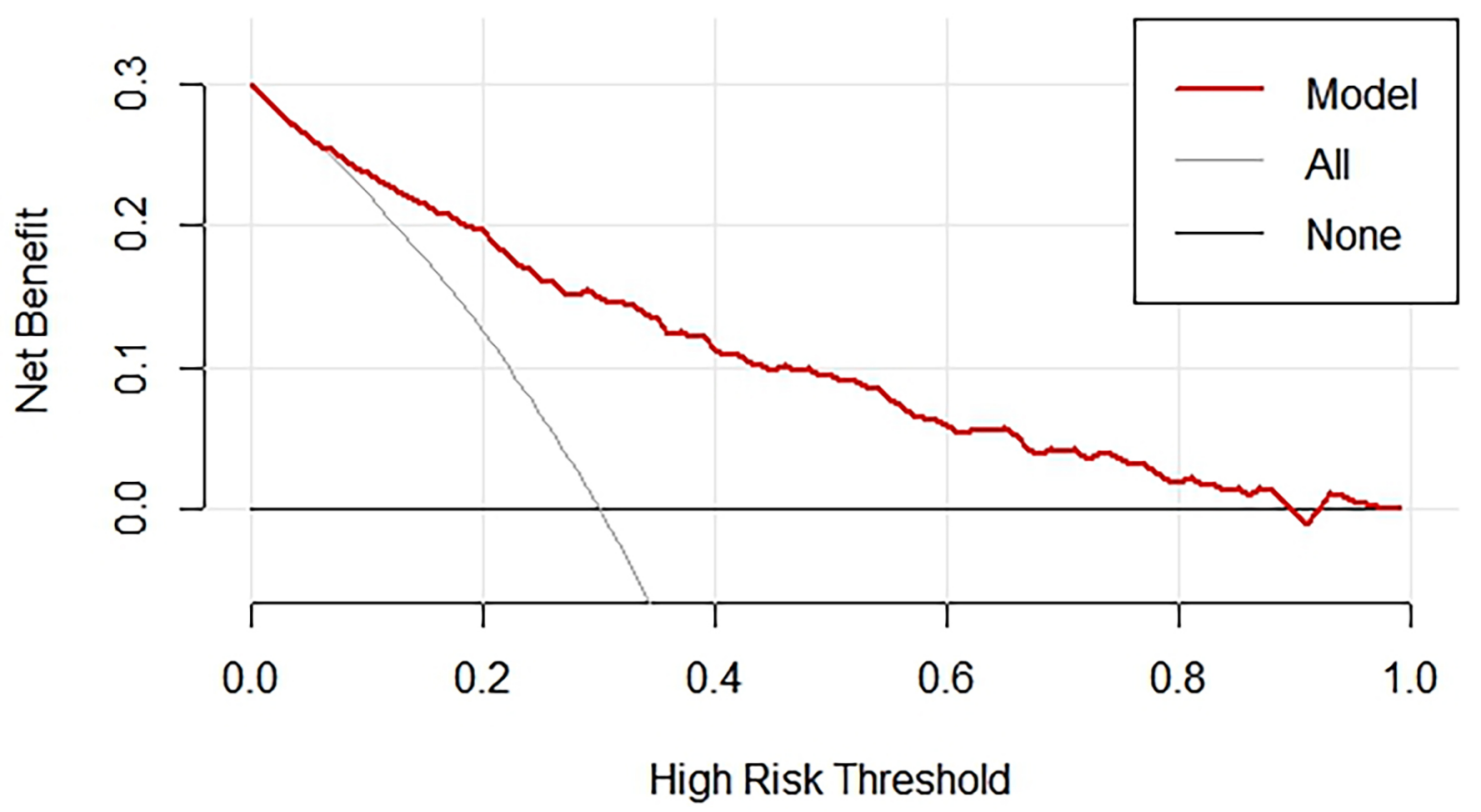
**DCA for nomogram validation**. DCA, decision curve analysis.

The presents a clinical DCA (Fig. 6), a tool used to evaluate the clinical utility of the nomogram model for predicting the presence of CCC. The graph shows the trend of net benefit of the model changes as the threshold probability for defining high risk increases. The red line, representing the nomogram, shows a decreasing net benefit as the high-risk threshold increases. This indicates that as the criteria for intervention become more stringent, the ability of the model to provide a net benefit diminishes. For comparison, the gray line, labeled “all”, shows the net benefit if all patients were classified as high risk, while the black line labeled “none” represents the net benefit if no patients were classified as high risk. These benchmark lines help assess the performance of the nomogram against these extreme scenarios.

## 4. Discussion

This study aims to investigate the clinical, angiographic, and laboratory parameters associated with the presence of CCC in patients with acute STEMI. Relevant variables were meticulously selected using LASSO, univariate, and multivariate logistic regression analyses, ensuring accuracy by excluding non-essential factors. By considering a wide range of clinical variables, it covers common and significant predictors, enhancing its applicability across diverse scenarios. The included variables including history of CHD, osmolality, fibrinogen levels, occlusions in the LAD, LCX, and RCA, and the Gensini score—are readily obtainable in clinical settings, ensuring both practicality and ease of use. Additionally, the straightforward nature of the nomogram allows clinicians to quickly assess risk without complex computations, facilitating its integration into routine practice. This makes the nomogram a valuable tool for predicting CCC in STEMI patients, supporting effective risk assessment and management.

The variables included in the nomogram are crucial for understanding and predicting the development of CCC. A history of CHD often indicates chronic ischemic conditions that stimulate collateral vessel formation as a compensatory mechanism to improve blood flow [13]. Osmolality affects vascular tone and endothelial function, affecting the coronary microenvironment and potentially impacting CCC formation by altering the balance between vasodilators and vasoconstrictors [14]. Fibrinogen levels, as markers of inflammation and coagulation, can indicate a pro-inflammatory state that might either promote or inhibit collateral development, depending on the balance of pro-angiogenic and anti-angiogenic factors [15]. Occlusions in major coronary arteries like the LAD, LCX, and RCA trigger collateral vessel development as the body attempts to bypass blockages and maintain myocardial perfusion [16]. The Gensini score quantifies the severity of coronary artery disease, with higher scores indicating more severe disease that can stimulate CCC as the heart seeks to compensate for reduced blood flow [17]. These variables are readily obtainable in clinical practice, ensuring the nomogram’s practicality and usability, allowing clinicians to effectively assess the likelihood of CCC development and aid in patient management and treatment planning [18].

When constructing a nomogram for predicting CCC, several important factors may be omitted for logical reasons. First, some indicators are excluded because they are not commonly used in current clinical practice, often due to high costs or limited availability. Second, treatments universally applied to all patients lack discriminatory power and are therefore not useful for inclusion. For example, pharmacological interventions such as statins and antiplatelet agents did not yield a significant result in the logistic regression model, as all patients in this study received these treatments. In fact, dual antiplatelet therapy, which typically involves aspirin and a P2Y12 inhibitor, prevents platelet aggregation and reduces vascular inflammation, thereby enhancing endothelial function [19]. Similarly, statins, known for their lipid-lowering effects, improve endothelial function by increasing nitric oxide bioavailability and reducing oxidative stress and inflammation [20]. These effects create a favorable environment for angiogenesis and stabilize atherosclerotic plaques, indirectly supporting collateral vessel development.

In the presence of CCC, the diagnostic criteria for STEMI remain based on electrocardiogram (ECG) and coronary angiography results. STEMI can be diagnosed after the onset of symptoms if there is significant ST-segment elevation is observed on the EGG and coronary angiography confirms the culprit artery corresponding to the EGG changes. ST-segment changes are dynamic; some patients may experience persistent elevation, while others may show a gradual decrease, which is associated with the formation of collateral circulation [21]. In cases of chronic or acute myocardial ischemia, collateral circulations can form differently depending on the individual coronary anatomy and the location of the obstructive lesions. In STEMI patients, collateral circulation typically involves other well-perfused coronary vessels that supply blood to the artery experiencing significant stenosis or occlusion. In the context of STEMI, the formation of CCC is particularly critical due to the acute and severe nature of the blockage Acute ischemia from STEMI increases shear stress, a potent stimulus for collateral vessel recruitment. Hypoxia in the affected myocardial tissue triggers the expression of hypoxia-inducible factors, which promote angiogenesis [22]. The inflammatory response following myocardial infarction enhances CCC development through the release of cytokine and growth factors. Pre-existing collaterals may rapidly enlarge during STEMI, providing immediate relief to the ischemic myocardium. Well-developed CCC can serve as a marker for better prognosis, potentially allowing for more conservative management and influencing the intensity of monitoring and follow-up care. It can also impact revascularization decisions, where patients with robust collateral networks might benefit from delayed or selective revascularization, choosing between PCI and coronary artery bypass grafting (CABG) [23]. Additionally, the presence of CCC may affect pharmacological therapy choices, as patients with well-developed CCC could respond differently to antiplatelet or anticoagulant treatments, enabling adjustments to optimize outcomes. Understanding CCC is also essential for anticipating and managing complications such as arrhythmias or heart failure, with those having poor CCC requiring more aggressive interventions. Furthermore, patients with well-developed CCC might receive different counseling on lifestyle modifications and long-term management strategies, focusing on maintaining and enhancing collateral growth through exercise and other interventions [24].

### Limitation

The observational design of this study limits its ability to establish causality between the identified predictors and CCC development. To mitigate potential biases, we ensured the accuracy of historical data by cross-referencing multiple sources and using standardized data collection procedures. However, the sample size may not be large enough to fully capture the diversity of the patient population, potentially limiting the generalizability of the findings. Additionally, the relatively short follow-up period may not adequately reflect long-term outcomes or the evolution of CCC. Variability in laboratory parameters due to differences in testing methods and patient conditions at the time of admission may affect the reliability of the associations identified. Conducted at a single institution, the findings may reflect local practices that do not represent broader populations. These limitations highlight the need for further research to validate and expand upon these findings, ideally incorporating multicenter data and longer follow-up periods to enhance their robustness and applicability.

## 5. Conclusions

In conclusion, our study constructed a nomogram that incorporates a history of CHD, osmolality, levels of fibrinogen, and LAD, LCX, and RCA closures, as well as the Gensini score to predict the development of CCC in patients with STEMI.

## Availability of Data and Materials

The datasets generated and analysed during the study are not publicly available as per the ethical approval for the study, but are available from the corresponding author on reasonable request.

## Supplementary Material

Supplementary material associated with this article can be found, in the online version, at https://doi.org/10.31083/RCM26477.
