# Supplementary material for: Predictors of Coronary Collateral Circulation in Patients with Acute ST-segment Elevation Myocardial Infarction: A Nomogram-based Approach
Source: Rev Cardiovasc Med. 2025 Apr 16;26(4):26477. doi: 10.31083/RCM26477 (PMC12059742; doi:10.31083/RCM26477)
Supplement: Supplementary file 1 [file 2153-8174-26-4-26477-s1.doc]

**Supplementary materials**

The study reveals several significant differences between the CCC and non-CCC groups. The CCC group had a higher incidence of old myocardial infarction (7.78% vs. 2.99%, P = 0.014) and elevated high sensitivity C-reactive protein levels (P = 0.001), indicating increased inflammation. Coagulation profiles also differed, with the CCC group showing higher international normalized ratio, D-dimer, and altered prothrombin time and activity. Thyroid function was notably different, with higher thyroxine and free T4 levels in the CCC group (P = 0.001 and P = 0.011, respectively). Additionally, the CCC group had lower mean corpuscular hemoglobin (P = 0.017) and fractional shortening (P = 0.006), suggesting differences in hematological parameters and cardiac function. The use of calcium channel blockers was less frequent in the CCC group (P = 0.031). This comprehensive patient information, along with additional data presented in Tables 1 and 2, provides a detailed overview of the clinical characteristics and laboratory findings. The variables that meet the inclusion criteria will be incorporated into the LASSO regression analysis, helping to identify the most relevant predictors and refine the model to better understand the factors associated with the presence of coronary collateral circulation (CCC) in this patient cohort.

Table 4. supplementary materials

| **Characteristic** | **non-CCC group, n = 501** | **CCC group, n= 167** | **P-value** |
| --- | --- | --- | --- |
| **Old** myocardial infarction (n,%) | 15 (2.99%) | 13 (7.78%) | 0.014 |
| **Years of diabetes** (years) | 9.25 (9.25, 9.25) | 9.25 (9.25, 9.25) | 0.558 |
| **Family history of early onset CHD** (n,%) | 11 (2.20%) | 1 (0.60%) | 0.313 |
| **Family history of ischemic stroke** (n,%) | 39 (7.78%) | 6 (3.59%) | 0.090 |
| **Family history of hemorrhagic stroke** (n,%) | 27 (5.39%) | 15 (8.98%) | 0.141 |
| **Glycated** hemoglobin (%) | 5.90 (5.50, 6.70) | 5.90 (5.50, 6.46) | 0.892 |
| **Glucose** (mmol/L) | 5.84 (4.99, 7.90) | 5.90 (4.96, 7.61) | 0.557 |
| **Systolic** blood pressure on admission (mmHg) | 124.19 ± 20.47 | 123.31 ± 20.47 | 0.630 |
| **Diastolic** blood pressure on admission (mmHg) | 72.00 (65.00, 80.00) | 72.00 (65.00, 82.50) | 0.847 |
| **Heart** rate (bpm) | 74.00 (65.00, 83.00) | 74.00 (66.00, 85.50) | 0.713 |
| **AST** (U/L) | 31.00 (21.00, 48.00) | 34.48 (24.00, 51.00) | 0.133 |
| **GFR** (mL/min/1.73 m²) | 84.97 (71.61, 96.73) | 82.15 (70.59, 95.99) | 0.347 |
| **High** sensitivity C-reactive protein（mg/L） | 6.51 (2.27, 13.24) | 9.93 (3.54, 19.13) | 0.001 |
| **Red** blood cells（10^12/L） | 4.41 ± 0.56 | 4.48 ± 0.61 | 0.218 |
| **Mean** corpuscular hemoglobin（pg） | 31.60 (30.50, 32.70) | 31.20 (30.11, 32.40) | 0.017 |
| **ESR**（mm/hr） | 12.00 (6.50, 21.00) | 14.00 (7.00, 25.00) | 0.101 |
| **NLR**（ratio） | 4.13 (2.72, 6.24) | 4.30 (2.67, 6.41) | 0.542 |
| **Absolute** lymphocyte count（10^9/L） | 1.60 (1.18, 2.04) | 1.63 (1.12, 2.12) | 0.842 |
| **Absolute** neutrophil count（10^9/L） | 6.46 (4.87, 8.59) | 6.56 (5.14, 8.68) | 0.774 |
| **Antithrombin** III（%） | 87.64 (80.30, 95.60) | 87.64 (77.95, 96.40) | 0.623 |
| **Globulin(g/dL)** | 24.90 (22.20, 27.40) | 25.70 (23.30, 27.80) | 0.034 |
| **International** normalized ratio（ratio） | 1.02 (0.97, 1.05) | 1.03 (0.98, 1.07) | 0.021 |
| **Fibrinogen** degradation products（µg/mL） | 1.60 (1.10, 2.49) | 1.70 (1.15, 2.80) | 0.071 |
| **D-dimer**（mg/L） | 0.70 (0.50, 1.02) | 0.80 (0.50, 1.10) | 0.026 |
| **Prothrombin** time（seconds） | 11.60 (11.10, 12.10) | 11.70 (11.25, 12.30) | 0.036 |
| **Prothrombin** time activity（%） | 97.35 (89.00, 105.90) | 95.80 (87.45, 101.50) | 0.005 |
| **Activated** partial thromboplastin time（seconds） | 26.60 (24.00, 28.50) | 26.90 (24.65, 30.35) | 0.038 |
| **Thyroxine**（µg/dL） | 78.92 (69.32, 90.29) | 83.31 (72.87, 99.00) | 0.001 |
| **Free** T4（ng/dL） | 0.87 (0.77, 1.00) | 0.91 (0.80, 1.05) | 0.011 |
| **Calcium**（mg/dL） | 2.13 (2.05, 2.21) | 2.12 (2.02, 2.21) | 0.191 |
| **Phosphorus**（mg/dL） | 1.08 (0.92, 1.21) | 1.04 (0.91, 1.17) | 0.164 |
| **Sodium**（mmol/L） | 140.30 (138.10, 142.30) | 139.90 (137.80, 141.80) | 0.182 |
| **Potassium**（mmol/L） | 4.11 (3.88, 4.34) | 4.13 (3.92, 4.38) | 0.220 |
| **Chloride**（mmol/L） | 102.54 (100.00, 104.90) | 101.90 (99.00, 103.55) | 0.001 |
| **Carbon** dioxide（mmol/L） | 23.94 ± 2.41 | 23.94 ± 2.67 | 0.965 |
| **Anion** gap（mmol/L） | 15.34 ± 3.37 | 15.96 ± 3.30 | 0.040 |
| **Dyslipidemia(n,%)** | 196 (39.12%) | 65 (38.92%) | 1.000 |
| **Glycated albumin value(%)** | 13.80 (12.60, 16.30) | 15.00 (13.30, 17.07) | 0.040 |
| **Stroke history(n,%)** | 56 (11.18%) | 20 (11.98%) | 0.888 |
| **Years of hypertension**（years） | 13.52 (10.00, 13.52) | 13.52 (10.00, 13.52) | 0.714 |
| **Years of coronary heart disease**（Years） | 6.72 (6.72, 6.72) | 6.72 (6.72, 6.72) | 0.355 |
| **Family history of coronary heart diseas**（n,%） | 137 (27.35%) | 51 (30.54%) | 0.487 |
| **Family history of hypertension**（n,%） | 115 (22.95%) | 42 (25.15%) | 0.635 |
| **Family history of high cholesterol**（n,%） | 1 (0.20%) | 0 (0.00%) | 1.000 |
| **Family history of diabetes**（n,%） | 45 (8.98%) | 15 (8.98%) | 1.000 |
| **Height** （m） | 1.69 (1.63, 1.73) | 1.68 (1.62, 1.72) | 0.385 |
| **Weight** （kg） | 70.00 (63.00, 80.00) | 75.00 (65.00, 80.00) | 0.080 |
| **ALP(U/L)** | 74.00 (63.00, 87.00) | 75.00 (65.00, 86.00) | 0.674 |
| **A/G ratio(ratio)** | 1.50 (1.32, 1.68) | 1.43 (1.28, 1.58) | 0.002 |
| **Pulse**（bpm） | 74.00 (65.00, 82.00) | 74.00 (66.00, 85.00) | 0.630 |
| **GGT**（U/L） | 25.00 (17.00, 38.00) | 29.00 (17.00, 46.00) | 0.117 |
| **Total protein**（g/dL） | 62.04 ± 5.27 | 62.35 ± 5.98 | 0.527 |
| **Albumin**（g/dL） | 36.90 (34.90, 39.10) | 36.30 (34.00, 39.00) | 0.052 |
| **Total bilirubin**（µmol/L ） | 12.65 (9.67, 16.43) | 13.56 (9.91, 17.45) | 0.177 |
| **Direct bilirubin**（µmol/L ） | 2.65 (1.89, 3.57) | 2.94 (1.98, 3.83) | 0.211 |
| **Indirect bilirubin**（µmol/L） | 9.80 (7.59, 13.10) | 10.82 (7.43, 13.81) | 0.184 |
| **Cholinesterase**（µmol/L） | 7.93 (6.92, 8.99) | 7.86 (6.47, 9.03) | 0.249 |
| **Uric acid**（µmol/L） | 319.00 (264.00, 375.00) | 330.95 (259.00, 380.00) | 0.434 |
| **Antianginal drugs on admission**（n,%） | 53 (10.58%) | 23 (13.77%) | 0.325 |
| **White blood cells**（10^9/L） | 8.74 (7.10, 10.82) | 8.70 (7.32, 10.91) | 0.888 |
| **Absolute** monocyte count（10^9/L） | 0.30 (0.18, 0.44) | 0.29 (0.18, 0.43) | 0.763 |
| **Absolute** eosinophil count（10^9/L） | 0.11 (0.08, 0.16) | 0.11 (0.08, 0.16) | 0.445 |
| **Absolute** basophil count（10^9/L） | 0.03 (0.02, 0.05) | 0.04 (0.02, 0.06) | 0.013 |
| **Hemoglobin**（g/dL） | 141.00 (128.00, 151.00) | 141.00 (128.00, 151.00) | 0.862 |
| **Hematocrit**（%） | 41.60 (38.40, 44.90) | 41.70 (38.20, 45.45) | 0.613 |
| **Mean corpuscular hemoglobin concentratio**（g/dL） | 336.00 (328.00, 344.00) | 335.00 (326.00, 342.50) | 0.261 |
| **Mean platelet volume**（fL） | 8.30 (7.70, 9.10) | 8.40 (7.80, 9.00) | 0.287 |
| **Red** cell distribution width SD（fL） | 12.90 (12.00, 13.80) | 13.10 (12.09, 13.90) | 0.135 |
| **Platelets**（10^9/L） | 225.00 (186.00, 267.00) | 219.00 (178.00, 261.50) | 0.403 |
| **Mean** corpuscular volume（fL） | 94.00 (91.00, 97.00) | 93.00 (90.50, 96.94) | 0.091 |
| **Platelet crit**（%） | 0.19 (0.16, 0.22) | 0.19 (0.15, 0.22) | 0.691 |
| **Platelet** volume distribution width（%） | 14.00 (12.30, 15.80) | 14.25 (12.50, 15.80) | 0.402 |
| **Thyroid** uptake rate（%） | 42.83 (41.30, 44.20) | 43.00 (41.25, 44.40) | 0.421 |
| **Thyroid** stimulating hormone（µIU/mL） | 1.14 (0.63, 1.91) | 1.07 (0.65, 1.96) | 0.71 |
| **Triiodothyronine**（ng/dL） | 74.92 (66.20, 85.53) | 76.14 (65.12, 86.49) | 0.633 |
| **Free T3**（pg/mL） | 2.59 (2.37, 2.80) | 2.56 (2.36, 2.82) | 0.809 |
| **SV(mm)** | 72.94 (64.44, 84.47) | 74.89 (67.28, 83.49) | 0.495 |
| **RV(mm)** | 1.60 (1.40, 1.80) | 1.60 (1.50, 1.80) | 0.133 |
| **Tricuspid** regurgitation（m/s） | 236.78 (211.00, 259.00) | 236.78 (211.00, 260.50) | 0.588 |
| **FS**（%） | 0.31 (0.27, 0.35) | 0.30 (0.24, 0.35) | 0.006 |
| **RV**（mm） | 1.60 (1.40, 1.80) | 1.60 (1.50, 1.80) | 0.133 |
| **E** wave（cm/s） | 78.00 (64.00, 92.50) | 76.00 (67.00, 90.00) | 0.972 |
| **A** wave（cm/s） | 85.00 (72.00, 96.00) | 85.76 (71.00, 101.50) | 0.165 |
| **EA** ratio | 0.91 (0.72, 1.20) | 0.86 (0.72, 1.16) | 0.516 |
| **Mitral** regurgitation（m/s） | 317.08 (256.00, 342.00) | 317.08 (279.50, 384.00) | 0.027 |
| **AV** forward（m/s） | 125.00 (110.00, 140.00) | 123.00 (107.00, 138.00) | 0.208 |
| **Antianginal drugs(n,%)** | 280 (55.89%) | 95(56.89%) | 0.88 |
| **Dual antiplatelet therapy(n,%)** | 488 (97.41%) | 160(95.81%) | 0.122 |
| **Beta** blockers(n,%) | 366 (73.05%) | 133 (79.64%) | 0.111 |
| **ACEI**/ACEI/ARNI（n,%） | 339 (67.66%) | 109 (65.27%) | 0.635 |
| **CCB**（n,%） | 54 (10.78%) | 8 (4.79%) | 0.031 |
| **Dual** antiplatelet therapy(n,%) | 2.00 (2.00, 2.00) | 2.00 (2.00, 2.00) | 0.851 |
| **Diuretics**（n,%） | 38 (7.58%) | 19 (11.38%) | 0.174 |
| **Statins(n,%)** | 451 (90.02%) | 152 (91.02%) | 0.821 |

Abbreviations: CCC: Coronary Collateral Circulation; CHD: Coronary Heart Disease; AST: Aspartate Aminotransferase; GFR: Glomerular Filtration Rate; NLR: Neutrophil-to-Lymphocyte Ratio; ESR: Erythrocyte Sedimentation Rate; NT-proBNP: N-terminal pro b-type Natriuretic Peptide; CKMB: Creatine Kinase-MB peak; MYO: Myoglobin peak; TnI: Troponin I peak; IABP: Intra-Aortic Balloon Pump; FS: Fractional Shortening; ALP: Alkaline Phosphatase; GGT: Gamma-Glutamyl Transferase; A/G ratio: Albumin/Globulin ratio; CCB: Calcium Channel Blocker; ACEI: Angiotensin-Converting Enzyme Inhibitor; ARNI: Angiotensin Receptor-Neprilysin Inhibitor; MACE: Major Adverse Cardiac Events; LDL: Low-Density Lipoprotein; HDL: High-Density Lipoprotein; LDL-C: Low-Density Lipoprotein Cholesterol; HDL-C: High-Density Lipoprotein Cholesterol; TSH: Thyroid Stimulating Hormone; T3: Triiodothyronine; T4: Thyroxine; Free T3: Free Triiodothyronine; Free T4: Free Thyroxine; SV: Stroke Volume; RV: Right Ventricle; AV: Aortic Valve; EA ratio: E/A Ratio; BMI: Body Mass Index; ALT: Alanine Aminotransferase.
